# An Efficient Two-Stage Decoding Scheme for LDPC-CRC Concatenated Codes

**DOI:** 10.3390/e27090899

**Published:** 2025-08-25

**Authors:** Lingjun Kong, Haiyang Liu, Yuezhuang Shi, Jiacheng Miao

**Affiliations:** 1 Faculty of Network and Telecommunication Engineering, Jinling Institute of Technology, Nanjing 211169, China; kong@jit.edu.cn (L.K.); jiachengmiao1290@gmail.com (J.M.); 2 Institute of Microelectronics, Chinese Academy of Sciences, Beijing 100029, China; syuez@hotmail.com; 3 University of Chinese Academy of Sciences, Beijing 100049, China

**Keywords:** concatenated codes, low-density parity-check (LDPC) codes, cyclic redundancy check (CRC) code, two-stage decoding

## Abstract

In modern communication systems, the concatenation of a low-density parity-check (LDPC) code with a cyclic redundancy check (CRC) code is commonly used for error correction. In this paper, we propose a low-complexity two-stage scheme for decoding these codes using their concatenation structures. In the first stage, the traditional belief propagation (BP)-based iterative algorithm with a relative small maximum number of iterations is performed for decoding the LDPC code. If an LDPC codeword is obtained in this stage, the decoding process terminates. Otherwise, the second stage of the decoding process is performed, in which the guessing random additive noise decoding (GRAND) algorithm is applied to the CRC code. A list of information sequences satisfying the CRC check is obtained, each of which is then encoded to an LDPC codeword. The most likely codeword among them is the output of the decoding approach. The simulation results indicate that the proposed two-stage decoding approach can outperform the traditional BP-based iterative algorithm with a large maximum number of iterations. Moreover, the average complexity of the proposed approach is relatively low.

## 1. Introduction

The stringent demands imposed by next-generation communication systems underscore the importance of short error-correcting codes (ECCs). The rapid development of artificial intelligence (AI) and 6G communication systems has imposed unprecedented demands on ultra-low latency and ultra-high reliability, particularly in mission-critical applications such as autonomous vehicles, the industrial IoT, and real-time edge computing [1]. In such scenarios, short ECCs are often preferred due to their ultra-low decoding latency and significantly low hardware complexity achieved by minimizing memory requirements compared to long codes [2]. Moreover, with superior channel adaptability and deep AI-decoder integration, short codes emerge as a sustainable solution for stringent demands of 6G communication systems. However, short codes typically suffer from weak error correction capabilities, necessitating advanced decoding techniques to achieve near-optimal performance without excessive computational overhead [3,4,5,6].

Since their rediscovery, low-density parity-check (LDPC) codes have received a lot of research interest due to their remarkable error correction capabilities. It can be shown that long LDPC codes can achieve near Shannon-limit error correction performances under belief propagation (BP)-based iterative algorithms [7,8,9,10,11]. On the other hand, the LDPC coding scheme designed for 6G communication systems should support a short code length (about one hundred bits) in order to meet the ultra-low latency requirement. Unfortunately, there is a considerable gap between the BP decoding performance and the corresponding theoretical bound for short LDPC codes, see, e.g., [12,13]. Some research works [14,15,16] have designed methods for decoding short LDPC codes using the idea of the ordered statistic decoding (OSD) algorithm, which can be applied to any linear code with a given generator matrix. However, the OSD algorithm is not suitable for LDPC codes compared with other algebraic codes (e.g., BCH codes), since these codes are not represented by their generator matrices in general. As a consequence, designing effective decoding algorithms for short LDPC codes is still a challenging problem.

A cyclic redundancy check (CRC) code [17] is an error detection code widely used in communication and storage systems to verify data integrity. In modern communication systems, an LDPC code is commonly concatenated with a CRC code for error correction [18,19,20], which is called an LDPC-CRC concatenated code in this paper. In such a concatenation coding scheme, the information sequence is pre-coded by a CRC code and then is encoded by an LDPC code to obtain a transmitted codeword. Traditionally, the aim of such a scheme is to reduce the undetectable error rate of the system. Interestingly enough, it has been shown that the concatenation scheme can also facilitate the design of an effective decoding algorithm to improve the overall performance. To date, several research works have considered enhanced decoding methods for LDPC-CRC concatenated codes [21,22,23]. For instance, the work in [21] introduces an innovative multi-round BP decoding strategy with impulsive perturbation, which can effectively overcome the effects of trapping sets that often limit the performance of short codes. Furthermore, the work in [22] demonstrates an enhanced decoding approach by integrating the CRC into the OSD process. Meanwhile, the authors of [23] provide valuable insights into the performance–complexity trade-offs between regular and irregular codes under a CRC-aided hybrid decoding framework. This motivates a new decoding paradigm: instead of treating CRC merely as a post-decoding check, we can leverage it as an integral component of the decoding process.

In recent years, guessing random additive noise decoding (GRAND), a novel universal decoding approach proposed by Duffy et al. [24], has received a lot of attention. GRAND performs the guessing process by ranking all the possible noise sequences in descending order of their likelihoods and removing them sequentially from the received sequence. The process terminates if a codeword is obtained or if the maximum guessing number is reached. It can be shown that GRAND can achieve near optimal performance with reasonable decoding complexity, especially for short linear codes. To date, various extensions of GRAND have been provided, see, e.g., [25,26,27,28,29,30]. Among them, the ordered reliability bits GRAND (ORBGRAND) algorithm [26] is one of the most promising versions of GRAND since it introduces an effective method for scheduling the noise sequences.

In this paper, we propose an efficient decoding scheme for LDPC-CRC concatenated codes. Our scheme is motivated by the following observation: For most LDPC codes, the performance gain of BP-based iterative decoding is reduced as the maximum number of iterations increases. As an example, it has been shown that the performance gap of LDPC codes constructed from finite geometries is within 0.2 dB when the maximum number of iterations is set as 20 and 100, respectively; see, e.g., [31] (Figure 10.2). This observation allows us to design a complexity-aware decoding scheme that dynamically adapts decoding efforts, particularly in combination with CRC-aided strategies. Concretely speaking, we provide a decoding approach that contains two stages. In the first stage, we use the traditional iterative algorithm (e.g., the min-sum algorithm) with a relative small maximum number of iterations. The decoding process terminates if an LDPC codeword is obtained in this stage. Otherwise, we perform the decoding of the CRC code in the second stage. To be specific, we apply the GRAND algorithm to the CRC code in the concatenation scheme and obtain a list of information sequences that satisfy the CRC check. Afterwards, we encode each information sequence into an LDPC codeword and choose the most likely codeword as the decoding output. The simulation results indicate that the proposed decoding approach can outperform the traditional iterative algorithm with a large maximum number of iterations. The analysis results suggest that the average complexity of the proposed algorithm is relatively low. To our knowledge, this represents the first successful integration of GRAND with LDPC-CRC concatenation structures.

The rest of this paper is organized as follows. Section 2 presents the background knowledge. We provide the proposed decoding scheme for LDPC-CRC concatenated codes in Section 3. Section 4 presents the simulation results. Finally, Section 5 concludes this paper.

## 2. Background Knowledge

In this section, we present essential background knowledge related to this work. We first introduce the fundamentals of LDPC codes and their iterative decoding algorithms. Then, we provide an overview of CRC codes and the GRAND algorithm. All the codes considered in this work are binary codes.

### 2.1. LDPC Codes and Iterative Decoding

An [n,k] LDPC code C is a linear code specified by a sparse parity-check matrix $H=[h_{ji}]$ of size m×n . (Note that H may have redundant rows, so we have m≥rank(H)=n−k , where rank(H) is the rank of H over the binary finite field.) For convenience, we associate H with a bipartite graph (called the Tanner graph [32]) that has *m* check nodes and *n* variable nodes corresponding to the *m* rows and *n* columns of H , respectively. The *i*-th variable node $v_{i}$ is connected to the *j*-th check node $c_{j}$ if and only if entry $h_{ji}$ in H is 1. Define

(1) $$M\left(i\right)=\left\{j:h_{ji}=1\right\},\;1\leq i\leq n,$$

(2) $$N\left(j\right)=\left\{i:h_{ji}=1\right\},\;1\leq j\leq m.$$

Traditionally, an LDPC code can be decoded by BP-based iterative algorithms. The decoding process can be viewed as message passing and updating on the edges of the Tanner graph that describes the code. Here, we describe the min-sum algorithm (MSA), one of the most prevalent BP-based iterative algorithms.

Suppose $\gamma_{i}$ is the log-likelihood ratio (LLR) of the *i*-th bit, which can be calculated by

(3) $$\gamma_{i}=\log\left(\frac{P(y_{i}|x_{i}=0)}{P(y_{i}|x_{i}=1)}\right),$$

where $y=(y_{1},y_{2},\ldots ,y_{n})$ is the channel output sequence.

For the *k*-th iteration, let $\alpha_{i\to j}^{\left(k\right)}$ and $\beta_{j\to i}^{\left(k\right)}$ be the messages from $v_{i}$ to $c_{j}$ and from $c_{j}$ to $v_{i}$ , respectively. The MSA is performed as follows [33].

Initialization

Set k=0 and the maximum number of iterations, $K_{\max}$ . For each (i,j) such that $h_{ji}=1$ , set

(4) $$\alpha_{i\to j}^{\left(0\right)}=\gamma_{i}.$$

Step 1

For 1≤j≤m and each i∈N(j) ,

(5) $$\beta_{j\to i}^{\left(k\right)}=\prod_{i^{\prime }\in N\left(j\right)\setminus \left\{i\right\}}\mathrm{sign}\left(\alpha_{i^{\prime }\to j}^{\left(k-1\right)}\right)\cdot \min_{i^{\prime }\in N\left(j\right)\setminus \left\{i\right\}}\left|\alpha_{i^{\prime }\to j}^{\left(k-1\right)}\right|.$$

Step 2

For 1≤i≤n and each j∈M(i) ,

(6) $${\tilde{\gamma }}_{i}^{\left(k\right)}=\gamma_{i}+\sum_{j\in M(i)}\beta_{j\to i}^{\left(k\right)},$$

(7) $$\alpha_{i\to j}^{\left(k\right)}={\tilde{\gamma }}_{i}^{\left(k\right)}-\beta_{j\to i}^{\left(k\right)}.$$

Step 3

Create the hard-decision vector ${\hat{x}}^{\left(k\right)}=\left({\hat{x}}_{1}^{\left(k\right)},{\hat{x}}_{2}^{\left(k\right)},\ldots ,{\hat{x}}_{n}^{\left(k\right)}\right)$ such that

(8) $${\hat{x}}_{i}^{\left(k\right)}=\left\{\begin{array}{cc} 1, & \mathrm{if}\;{\tilde{\gamma }}_{i}^{\left(k\right)}\leq 0,\\ 0, & \mathrm{if}\;{\tilde{\gamma }}_{i}^{\left(k\right)}>0. \end{array}\right.$$

Step 4

If ${\hat{x}}^{\left(k\right)}H^{⊤}=0$ holds or $k=K_{\max}$ , stop the iteration process and output ${\hat{x}}^{\left(k\right)}$ as the decoding result. Otherwise, set k←k+1 and go to Step 1.

### 2.2. CRC Codes and GRAND

A CRC code is a shortened cyclic code that is defined by a generator polynomial $g\left(x\right)=\sum_{i=0}^{r}g_{i}x^{i}$ .

Assume that an information sequence $u\left(x\right)=\sum_{i=0}^{k-1}u_{i}x^{i}$ of length *k* is transmitted. The CRC encoding procedure can be described as

(9) c(x)=u(x)g(x).

In this manner, we obtain a binary linear code with length k+r and dimension *k*.

The encoding procedure of a CRC code can also be performed under the following systematic manner

(10) $$c\left(x\right)=x^{r}u\left(x\right)+s\left(x\right),$$

where s(x) , the CRC polynomial of the information sequence, is given by

(11) $$s\left(x\right)=x^{r}u\left(x\right)\;\mathrm{mod}\;g\left(x\right).$$

The systematic encoding of a CRC code can be efficiently implemented using a shift register; see, e.g., [17] for details.

Compared with other linear codes, CRC codes have the advantage that the information length can be flexibly adjusted.

Suppose a codeword, c , of a binary linear code, C , with length *n* is transmitted. Let the hard-decision sequence from the channel be

(12) $$d=\left(d_{1},d_{2},\ldots ,d_{n}\right)\in {\left\{0,1\right\}}^{n}.$$

The GRAND algorithm operates by sequentially guessing noise sequences z introduced by the channel to recover the transmitted codeword. In other words, the decoder tries noises z such that

(13) $$\hat{c}=d⊕z,\;\mathrm{with}\;\hat{c}\in C.$$

GRAND implements a maximum-likelihood (ML) decoding rule by prioritizing noise patterns based on their a priori likelihoods. Under the common assumption that the noise sequence has the lowest Hamming weight, the ML decoding is expressed as

(14) $$\hat{c}=\arg\max_{c\in C}P\left(d|c\right)=\arg\max_{z}P\left(z\right)\;s.t.\;c=d⊕z.$$

In the classical GRAND scheme, candidate noise patterns are tested in ascending order of Hamming weights, i.e., noise patterns z with small

(15) $$w_{H}\left(z\right)=\sum_{i=1}^{n}z_{i}$$

are examined first, focusing decoding efforts on the most probable error patterns.

### 2.3. ORBGRAND

Ordered reliability bits guessing random additive noise decoding (ORBGRAND) refines the original GRAND framework by incorporating ordered bitwise reliability into the prioritization of candidate noise patterns. Instead of exhaustively testing noise sequences based solely on their combinatorial simplicity, ORBGRAND utilizes probabilistic ranking derived from soft reliability metrics, typically based on LLR values magnitudes, to guide decoding. This ordered strategy enables the decoder to explore more probable error configuration patterns first, which can significantly improve the decoding accuracy.

Let the received soft-decision channel output r be

(16) $$r=\left(r_{1},r_{2},\ldots ,r_{n}\right)\in R^{n}.$$

The hard-decision sequence d is

(17) $$\;d=\left(d_{1},\ldots ,d_{n}\right)\in {\left\{0,1\right\}}^{n},$$

where $d_{i}=\frac{1-\mathrm{sign}(r_{i})}{2}$ .

The bitwise reliability values, ϱ , are defined by LLR magnitudes

(18) $$\;\varrho =(\varrho_{1},\ldots ,\varrho_{n}),$$

where $\varrho_{i}=\left|\mathrm{LLR}\left(r_{i}\right)\right|$ .

A permutation, σ , is introduced such that

(19) $$\varrho_{\sigma (1)}\leq \varrho_{\sigma (2)}\leq \ldots \leq \varrho_{\sigma (n)}.$$

Let $z\in {\left\{0,1\right\}}^{n}$ be a candidate error pattern defined over the reliability-sorted domain. To reflect the likelihood of each pattern, a logistic weight is assigned as

(20) $$\omega \left(z\right)=\sum_{i=1}^{n}i\cdot z_{i}.$$

The decoder proceeds by enumerating all patterns z such that ω(z)=μ for increasing values of $\mu \in Z_{\geq 0}$ , i.e.,

(21) $$E_{\mu }=\left\{z\in {\left\{0,1\right\}}^{n}:\omega \left(z\right)=\mu \right\}.$$

To implement the decoding process, ORBGRAND explores error patterns in ascending order of logistic weight. For each weight level μ , candidate patterns $z\in E_{\mu }$ are generated and evaluated sequentially. Each error pattern is then mapped back to the original bit order using the inverse permutation $\sigma^{-1}$ , and the decoded candidate is formed as

(22) $$x=d⊕\sigma^{-1}\left(z\right).$$

## 3. Proposed Two-Stage Decoding Scheme

### 3.1. Motivation

Traditionally, an LDPC code is decoded with BP-based algorithms that are performed in an iterative manner. It is hoped that the performance becomes better as the number of iterations increases. However, the known results indicate that the performance gain of BP-based iterative decoding is reduced as the maximum number of iterations increases for most LDPC codes with short to moderate lengths [31]. Moreover, as the signal-to-noise ratio (SNR) increases, the BP-based iterative decoding falls short of the optimal decoding. It is known that certain harmful structures, called trapping sets [34,35], in an LDPC matrix degrade the performance of BP-based iterative decoding. The identification of such structures and the performance prediction using the structures have received a lot of interest. For details, the reader can refer to [36,37,38,39].

On the other hand, an LDPC code is commonly concatenated with a CRC code in modern communication systems. For such an LDPC-CRC concatenated code, the information sequence is pre-coded by a CRC code and then is encoded by an LDPC code to obtain a codeword. Traditionally, we perform LDPC decoding at first and then perform CRC check if the decoding is successful. The CRC check can detect residual errors after LDPC decoding and reduce the undetectable error rate of LDPC decoding. (An LDPC decoding process is said to be successful if a codeword is obtained. However, the obtained codeword may not be the transmitted codeword. In this case, an undetectable error of LDPC decoding occurs.)This is especially helpful when the LDPC code has short to moderate lengths. Recently, it has been shown that CRC codes can be efficiently decoded by GRAND algorithms when these codes are used for error correction [20]. This motivates us to design a two-stage decoding algorithm to compensate for the above-mentioned problem in BP-based iterative decoding.

### 3.2. Algorithm Design

As mentioned in the previous subsection, the proposed algorithm contains two stages. The traditional BP-based iterative algorithm is used to decode the LDPC code. The maximum number of iterations of LDPC decoding is set as a relatively small number, which is due to the aforementioned fact that performance improvement is limited when the number is increased to a certain value.

If the decoding is successful, i.e., an LDPC codeword is obtained, we terminate the decoding process and perform a CRC check. Otherwise, we consider the decoding of CRC codes as error correction codes. We apply the GRAND algorithm to the CRC code in the concatenation scheme and obtain a list of CRC codewords that can satisfy the CRC check. Finally, we encode each obtained CRC codeword to an LDPC codeword and choose the most likely codeword as the decoding output. If no CRC codeword is found, our decoding approach fails. In this case, we use the LDPC decoding result in the first stage as the decoding output.

In order to achieve desirable performances, the size of listed CRC codewords should be reasonably large. This is equivalent to setting a relatively large number of queries in the GRAND algorithm, which inevitably increases GRAND’s complexity. However, it is worth mentioning that the GRAND algorithm is performed only when LDPC decoding in the first-stage decoding fails. This indicates that the total complexity of the proposed two-stage decoding scheme is reasonable, especially when the SNR is moderate. The complexity analysis of the proposed decoding scheme is provided in the subsequent subsection.

**Figure 1:**
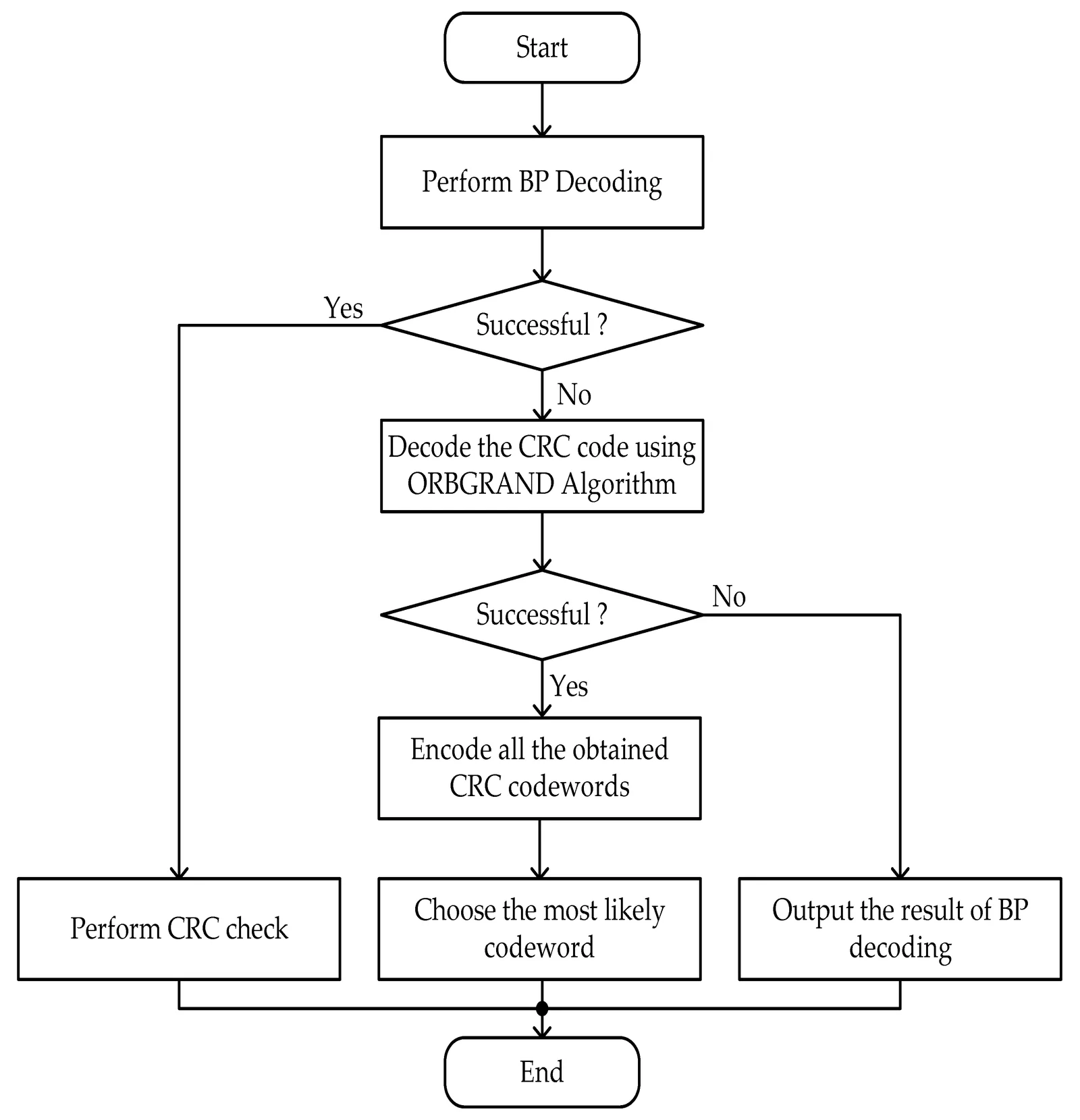
A flowchart of the proposed approach.

Overall, the flowchart of the proposed decoding approach is provided in Figure 1.

### 3.3. Complexity Analysis

In order to measure the complexity of the proposed two-stage decoding scheme, we use the number of operations required for decoding a received vector. This number can be derived based on the algorithm description in the previous subsection as well as the related references. For ease of analysis, we assume that the LDPC code is a (γ,ρ) -regular code described by an m×n parity-check matrix in the following. The results can be generalized to irregular LDPC codes in a straightforward manner.

#### 3.3.1. BP Decoding Complexity

Suppose the MSA is used for BP decoding an LDPC code. Let *I* be the average number of iterations. According to [40], we need $N_{A}=2n\gamma -n$ real additions, $N_{C}=m\left(\rho +\lceil \log_{2}\rho \rceil -2\right)$ real comparisons, and $N_{X}=2m\rho -m$ binary additions (XORs) in one iteration. Then, BP decoding complexity can be estimated as

(23) $$C_{\mathrm{BP}}=I\cdot \left(N_{A}+N_{C}+N_{X}\right).$$

#### 3.3.2. GRAND Complexity

Suppose the CRC code is specified by a parity-check matrix of size r×m . The complexity $C_{\mathrm{GRAND}}$ of GRAND consists of two parts:

1. Initial sorting requires $N_{S}=\frac{m(m+1)}{2}$ real comparisons to obtain the bit reliability order.
2. Error pattern testing requires binary operations. For an error pattern of Hamming weight *t*, at most rt , binary operations are needed. Hence, the complexity $N_{P}$ of error pattern testing is upper bounded by $Mr\bar{t}$ , where *M* is the number of tested error patterns, and $\bar{t}$ is the average Hamming weight of tested error patterns.

It is worth mentioning that the actual number of binary operations can be reduced if an appropriate scheduling of error patterns is used [41].

#### 3.3.3. LDPC Encoding Complexity

Richardson and Urbanke [42] pioneered the first efficient encoding algorithm for random LDPC codes. Their analysis suggested that gap parameter *g* scales as $O(\sqrt{n})$ for random codes but remains sufficiently small in practice. Consequently, the online encoding complexity $C_{\mathrm{enc}}$ reduces to $O(n+g^{2})$ (see [42] for details). For quasi-cyclic (QC) LDPC codes, the encoding complexity, $C_{\mathrm{enc}}$ , attains O(n) through shift register implementations [43,44].

#### 3.3.4. Proposed Scheme Complexity

The complexity of the proposed two-stage decoding scheme, denoted by $C_{\mathrm{prop}}$ , can be estimated as

(24) $$C_{\mathrm{prop}}=C_{\mathrm{BP}}+\alpha \left(C_{\mathrm{GRAND}}+C_{\mathrm{enc}}\right),$$

where α is the probability of the second stage of decoding. Since the second stage of decoding is invoked only when the first stage of BP decoding does not find any valid codeword, parameter α is upper bounded by the block error rate (BLER) of LDPC decoding in the first stage. (Note that LDPC decoding errors contain both detectable errors and undetectable errors. If an undetectable error occurs, the second stage of decoding is not invoked.)

In typical channel conditions, the BLER of LDPC decoding is below $10^{-1}$ . (For example, as shown in the next section, the BLER of a random (3,15)-regular LDPC code at 5 dB is about 0.05, where the MSA is used and the maximum number of iterations is set to 15). This indicates that most decoding tasks can be resolved in the first stage of decoding, and only a small fraction requires the second stage of decoding. Hence, the extra decoding complexity introduced by the proposed scheme is relatively low.

## 4. Simulation Results

In this section, we evaluate the performance of the proposed decoding scheme. For comparison purposes, the existing perturbation decoding scheme [21] for LDPC-CRC concatenated codes is also evaluated. We assume binary phase-shift keying (BPSK) modulation over the additive white Gaussian noise (AWGN) channel in our simulation. A regular [110,88] random LDPC code and a regular [169,132] QC-LDPC code are used. Each code is concatenated with a CRC-16 code, and the resulting schemes are denoted as $C_{1}$ and $C_{2}$ , respectively. The parity-check matrix of the LDPC code in $C_{1}$ is constructed based on the progressive-edge growth method [45], and the parity-check matrix of the LDPC code in $C_{2}$ is an array-based QC matrix [46]. The parameters of the two LDPC-CRC concatenated codes are listed in Table 1.

The decoding schemes evaluated include the following:

- MSA15-GRAND ζ : The proposed two-stage decoding scheme, where the maximum number of iterations in the standard MSA is set to 15. Parameter ζ∈{16,19,20} specifies the maximum number of error patterns tested (i.e., $2^{\zeta }$ ) in the GRAND algorithm.
- MSA15-NOISE: A two-stage decoding scheme where the standard MSA, limited to 15 iterations, is followed by noise-perturbed decoding, whose maximum number of decoding attempts is set to 30. The noise variance is optimized at a given SNR. For details, the reader can refer to [21].
- MSA15: The standard MSA with a maximum of 15 iterations.
- MSA50: The standard MSA with a maximum of 50 iterations.

**Figure 2:**
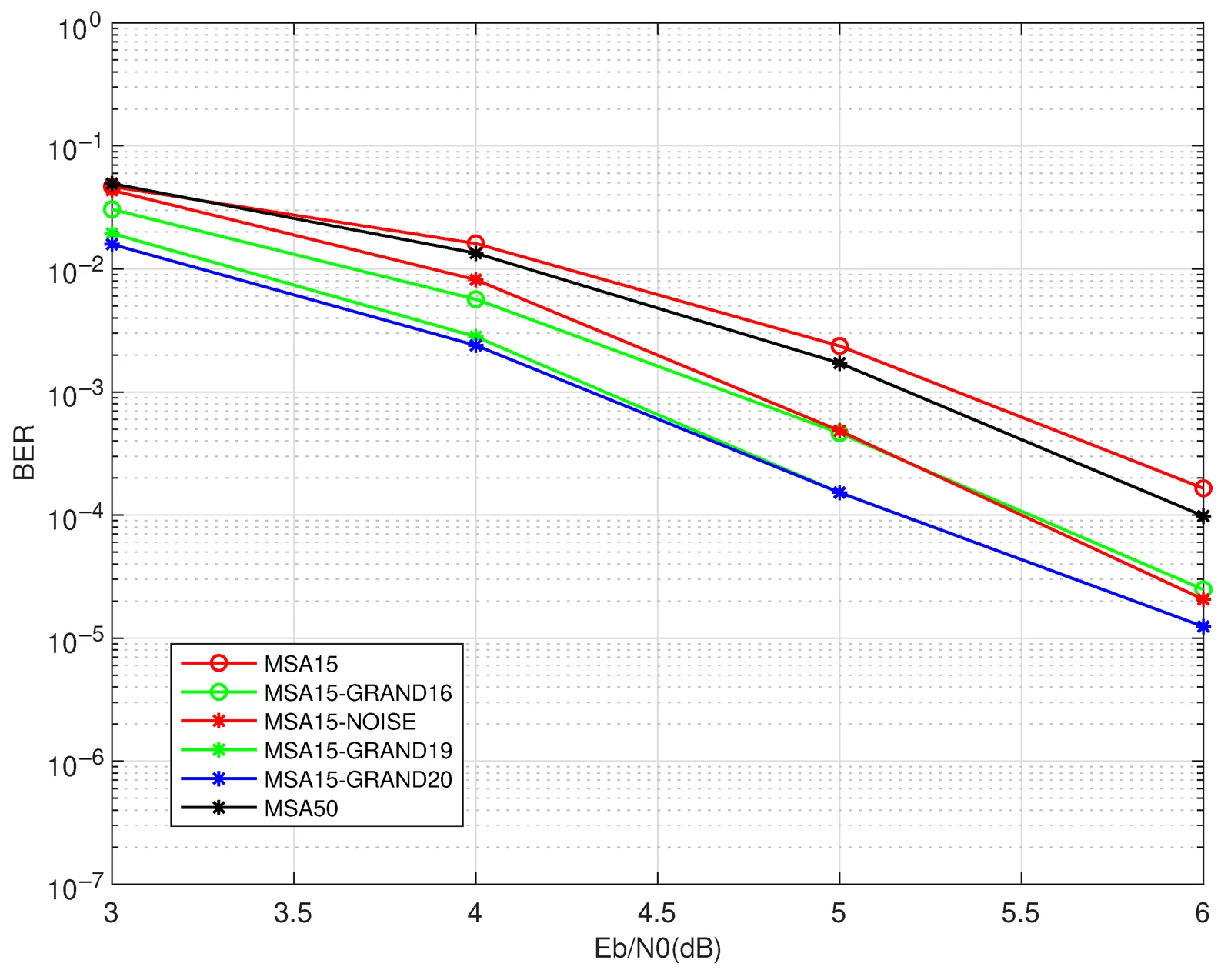
BER performance of different decoding schemes for $C_{1}$ .

As shown in Figure 2, increasing the maximum number of iterations in the MSA from 15 to 50 yields only a marginal improvement in the BER performance for $C_{1}$ . This observation suggests that merely increasing the maximum number of iterations does not necessarily lead to significant performance.

In contrast to the limited improvement observed with increased MSA iterations, the proposed two-stage decoding schemes (MSA15-GRAND ζ ) significantly outperform both MSA15 and MSA50 within the simulated SNR range. As ζ increases from 16 to 20, the BER performance improves accordingly, demonstrating a controllable trade-off between the decoding complexity and performance. Notably, MSA15-GRAND20 achieves a better performance than MSA50, despite using a lower number of MSA iterations, confirming the effectiveness of the GRAND-enhanced post-processing stage.

Meanwhile, scheme MSA15-NOISE also outperforms MSA15, although the gain is less significant compared to the proposed two-stage decoding schemes (MSA15-GRAND ζ ). This suggests that while noise-perturbed decoding can offer performance improvements, its potential is limited compared to the systematic test pattern exploration adopted by GRAND.

**Figure 3:**
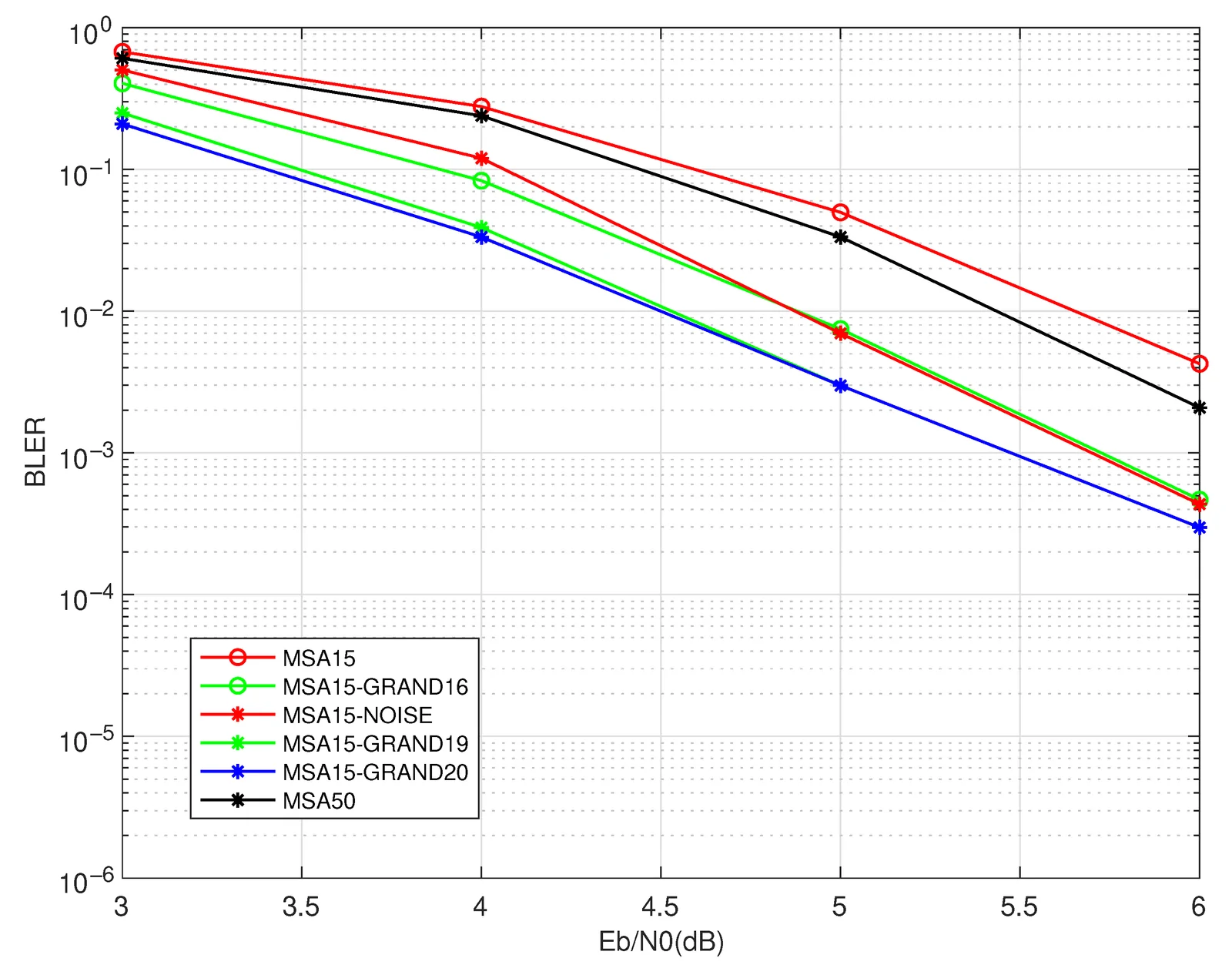
BLER performance of different decoding schemes for $C_{1}$ .

Figure 3 illustrates the BLER performance of various decoding schemes for $C_{1}$ , reflecting decoding reliability at the block level. While the overall trend resembles that of the BER performance in Figure 2, the impact of different decoding strategies on complete codeword recovery is more pronounced. In particular, the proposed MSA15-GRAND ζ schemes demonstrate significantly stronger block-level error correction, with MSA15-GRAND20 achieving up to an order-of-magnitude lower BLER compared to MSA50. This reinforces the suitability of GRAND-assisted decoding in systems requiring stringent block-level reliability. Although scheme MSA15-NOISE also reduces the BLER relative to the baseline, its improvements are less consistent, especially in the high-SNR regime.

**Figure 4:**
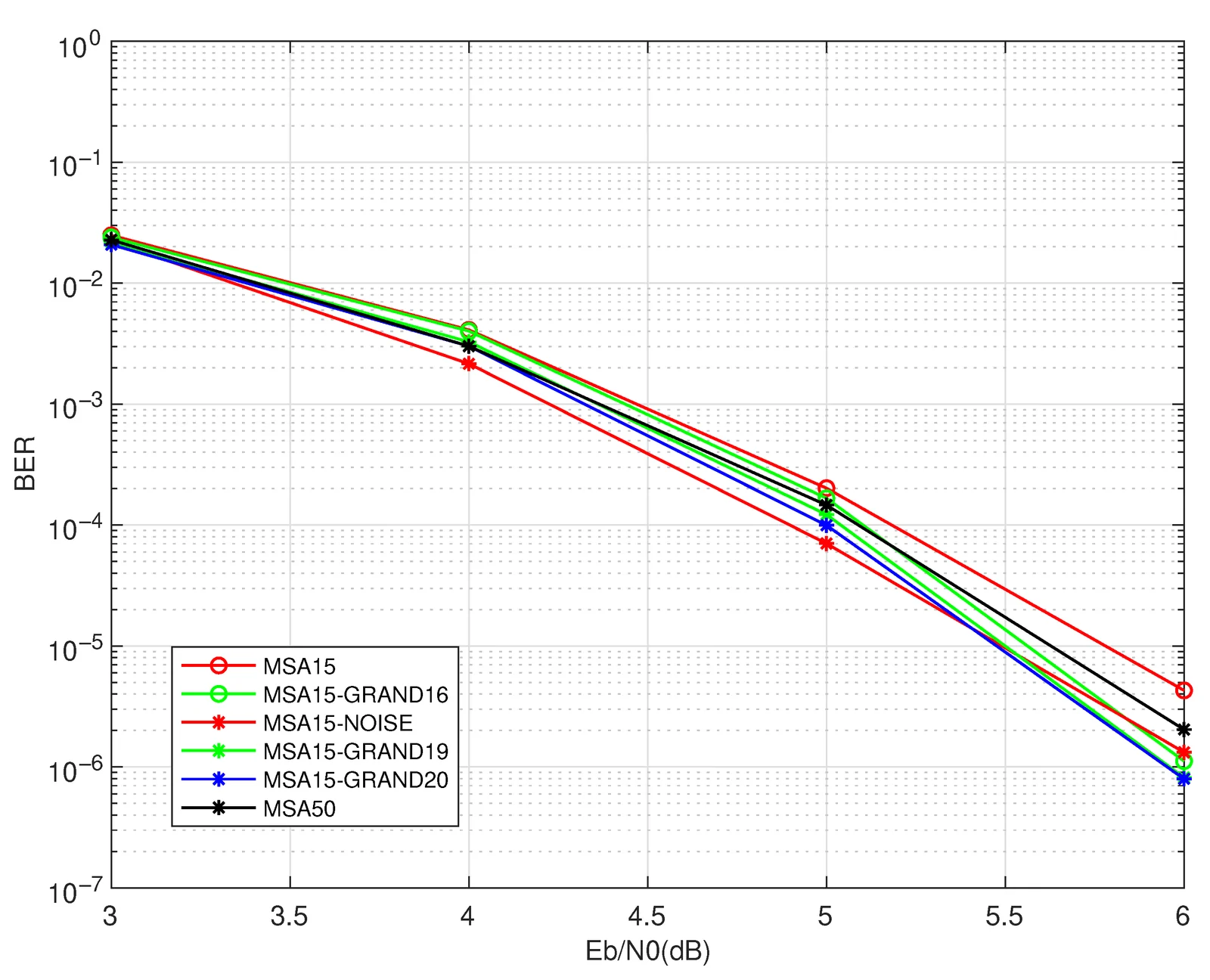
BER performance of different decoding schemes for $C_{2}$ .

**Figure 5:**
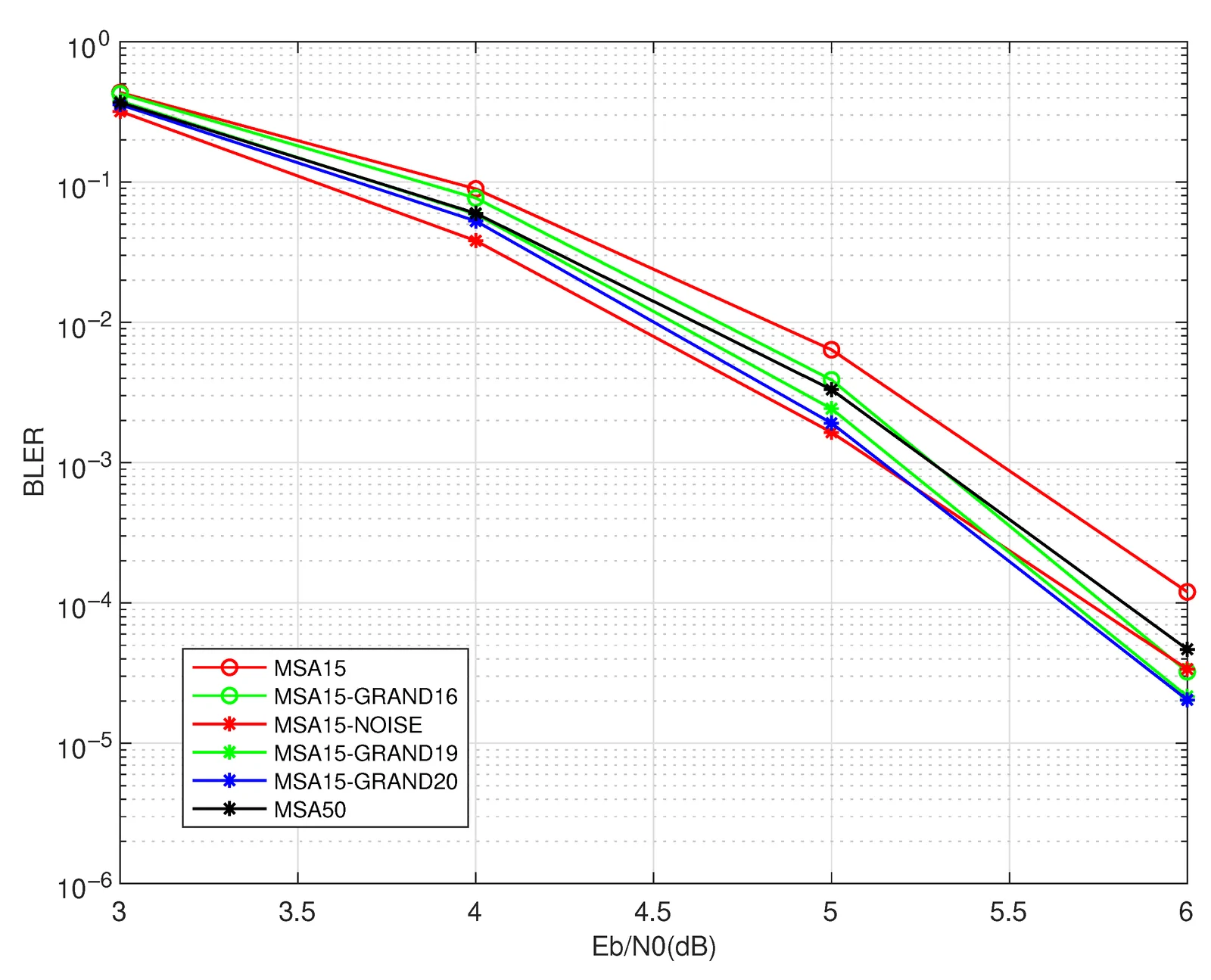
BLER performance of different decoding schemes for $C_{2}$ .

Figure 4 and Figure 5 illustrate the BER performance and the BLER performance of various decoding schemes applied to $C_{2}$ . Compared to the results observed in $C_{1}$ , all decoding schemes achieve a substantially better error performance within the simulated SNR range, highlighting the advantages of structured code construction.

In Figure 4, the proposed schemes MSA15-GRAND ζ continue to provide the best overall BER performance, particularly in the medium-to-high SNR region. Although the relative gains over MSA50 are smaller than those for $C_{1}$ , the improvement remains consistent and measurable. Interestingly, MSA15-NOISE shows competitive behavior in the low-SNR regime (i.e., SNR < 5.3 dB), temporarily outperforming all other schemes. This indicates that in highly noisy conditions, stochastic perturbations may offer early decoding benefits. However, as the SNR increases, its performance quickly saturates and is eventually surpassed by the proposed scheme.

Figure 5 presents the BLER performance of $C_{2}$ . The structured nature of $C_{2}$ again leads to a significantly reduced BLER compared to $C_{1}$ . The scheme MSA15-GRAND20 achieves the lowest BLER across most of the SNR range, confirming its effectiveness at the block level. The gain over MSA50, while modest, remains stable. Meanwhile, MSA15-NOISE maintains an edge in the low-SNR region but fails to preserve that advantage at the high-SNR region, suggesting a lack of robustness and generality.

Overall, the results for $C_{2}$ validate the general applicability of the proposed decoding scheme and demonstrate its advantages for QC-LDPC codes. Despite the narrow low-SNR advantage of MSA15-NOISE, schemes MSA15-GRAND ζ consistently deliver the best performance in practical operating regimes, offering an effective trade-off between complexity and reliability.

## 5. Conclusions

In this work, an efficient two-stage decoding approach was proposed for LDPC-CRC concatenated codes. The decoding process is designed by monitoring the convergence behavior of BP decoding. When BP decoding fails within a limited number of iterations, a secondary decoding stage is triggered, in which a CRC-guided GRAND algorithm is employed. The proposed approach can outperform BP decoding at the expense of relatively low extra decoding complexity. This balance between the decoding performance and complexity renders the proposed scheme suitable for latency-sensitive and resource-limited systems. Our future work will focus on integrating advanced GRAND pruning mechanisms and hybrid decoding strategies that incorporate perturbation theory and reliability-aware heuristics, with the specific aim of conducting a systematic performance and complexity comparisons with the OSD-based [22] and hybrid BP-ML [23] decoding frameworks.

## Figures and Tables

**Table 1:** Parameters of the two LDPC-CRC concatenated codes.

Code	*k*	*r*	( γ , ρ ) of LDPC Matrix	CRC Generator Polynomial
$C_{1}$	72	16	(3,15)	$x^{16}$ + $x^{12}$ + $x^{5}$ +1
$C_{2}$	116	16	(3,13)	$x^{16}$ + $x^{12}$ + $x^{5}$ +1

## Data Availability

The original contributions presented in this study are included in the article. Further inquiries can be directed to the corresponding author.
